# Prevalence of Obesity in Abdominal Surgery Patients at a Tertiary Care Hospital: A Cross-Sectional Study

**DOI:** 10.7759/cureus.74679

**Published:** 2024-11-28

**Authors:** Rehan Munir, Nizam Ul Haq, Babar Ali, Atif Iqbal, Kamran A Shah, Muhammad Usman Khan, Muhammad Umair

**Affiliations:** 1 General Surgery, Lady Reading Hospital, Peshawar, PAK; 2 General Surgery, Mardan Medical Complex, Mardan, PAK; 3 General Surgery, Jinnah International Hospital, Abbottabad, Abbottabad, PAK; 4 General Surgery, University Hospitals Birmingham NHS Foundation Trust, Birmingham, GBR; 5 General Surgery, Northwest General Hospital and Research Centre, Peshawar, PAK

**Keywords:** abdominal surgery, obesity, postoperative complications, prevalence, tertiary care hospital

## Abstract

Background

Obesity significantly impacts surgical outcomes, increasing the risk of postoperative complications, especially in abdominal surgery.

Objective

To determine the prevalence of obesity among patients undergoing abdominal surgery and to explore its association with postoperative complications.

Methodology

A cross-sectional study was conducted from January 2022 to December 2023. There were 428 adult patients in total, either for emergency or elective abdominal surgery. A BMI of ≥30 kg/m² was used by the World Health Organization to define obesity. We gathered and examined data on postoperative complications, surgical outcomes, comorbidities, and demographics. Chi-square and t-tests were used to assess the prevalence of obesity and its relationship to complications; a p-value of less than 0.05 was deemed statistically significant.

Results

Among 428 patients, 151 (35.28%) were obese. Obese patients had significantly higher rates of wound infections, with 36 (23.84%) compared to 28 (10.11%) non-obese patients (p = 0.001). Venous thromboembolism occurred in 18 (11.92%) obese patients versus nine (3.25%) non-obese patients (p = 0.002). Respiratory complications were more frequent in obese patients, with 27 (17.88%) compared to 31 (11.19%) non-obese patients (p = 0.045). Prolonged hospital stays (>7 days) were reported in 52 (34.44%) obese patients versus 39 (14.08%) non-obese patients (p < 0.001). Additionally, obesity was associated with longer surgery durations (124.35 ± 35.82 minutes in obese patients versus 108.65 ± 29.44 minutes in non-obese patients, p = 0.003) and extended recovery times (11.58 ± 5.67 days versus 8.73 ± 4.25 days, p = 0.002).

Conclusion

Patients having abdominal surgery are often obese, which is linked to an increased risk of complications and lengthier recovery times.

## Introduction

Obesity is a major worldwide health issue that has an influence on many facets of healthcare, including the results of surgeries [1]. It is defined by an excessive buildup of fat and is linked to a number of comorbidities, including diabetes, pulmonary problems, and cardiovascular disorders - all of which may affect surgery patients' outcomes and healing times [2]. Due to higher surgical risks, longer recovery periods, and the possibility of postoperative complications, abdominal surgery in obese individuals, in particular, poses specific concerns [3].

Obesity has become more common in recent years due to nutritional changes and lifestyle changes, such as sedentary behavior [4]. Healthcare systems are finding it difficult to handle the growing number of obese patients in many areas, which makes surgical treatment more complicated [5]. Patients who are obese and undergoing abdominal surgery are more likely to have wound infections, hernias, venous thromboembolism (VTE), and other problems that may increase the length of stay in the hospital and compromise surgical results in general [6]. Because of this, obesity is now taken into consideration during preoperative evaluations and surgery planning, especially in high-stakes settings like tertiary care facilities [7].

In order to improve clinical outcomes, tertiary care institutions often treat patients with a wide variety of comorbid diseases; thus, accurately determining the frequency of obesity in surgical populations is essential [8]. It will help improve preoperative planning, risk assessment, and postoperative care if surgical patients undergoing abdominal operations are aware of the unique issues that obesity presents [9]. Healthcare professionals might better predict issues and customize therapies to enhance surgical outcomes by knowing how common obesity is among these individuals [10].

Both internationally and in Pakistan, the incidence of obesity has increased to concerning proportions [11]. The World Health Organization reports that, since 1975, the incidence of obesity worldwide has quadrupled, accounting for 13% of adult obesity [12]. Despite the rise of metabolic bariatric surgeries such as laparoscopic Roux-en-Y gastric bypass (LRYGBP), the prevalence of obesity continues to increase, underscoring the severity of this global health issue. While bariatric surgery has demonstrated significant benefits in weight reduction and the remission of comorbidities such as type 2 diabetes mellitus (T2DM), its impact on controlling obesity prevalence on a population level remains limited. Kehagias et al. [13] found that modifying LRYGBP with fundus resection (LRYGBP + FR) did not improve glycemic control compared to standard LRYGBP, though it led to greater BMI reduction at 12 months. Despite the rise of metabolic bariatric surgery, the prevalence of obesity continues to increase, emphasizing the severity of this global health crisis and the need for broader public health interventions. Patients who are obese, especially those undergoing abdominal surgery, are more likely to have postoperative complications such as infections, breathing problems, and longer hospital stays [14]. Because obesity is becoming more common, surgical treatment is becoming more complex, which emphasizes the need for evaluating its effects in tertiary care settings [15].

This investigation, examining the frequency of obesity in patients undergoing abdominal surgery at a tertiary care facility, aims to provide light on the extent of this developing issue in a specialized medical context. The objective of this study was to determine the prevalence of obesity among adult patients undergoing abdominal surgery in a tertiary care hospital. While the primary focus was on obesity prevalence, its association with select surgical outcomes was explored as a secondary aim, recognizing that the analysis does not account for other potential influencing factors.

## Materials and methods

Study design and setting

This was a cross-sectional study conducted at the Department of Surgery, Lady Reading Hospital, Peshawar, Pakistan. The study was carried out over a period of two years, from January 2022 to December 2023.

Inclusion and exclusion criteria

All adult patients (age > 18 years) undergoing elective or urgent abdominal surgery throughout the study period met the inclusion criteria for the research. Patients with inadequate medical data, patients undergoing repeated abdominal operations over the research period, and pregnant women were excluded.

Sample size

To calculate the sample size for the study on obesity prevalence among surgical patients, we used the WHO formula for prevalence studies. The necessary sample size was determined to be around 385 people, assuming a 95% confidence level (Z = 1.96), an estimated prevalence of 50% (p = 0.5), and a 5% margin of error (E = 0.05). In order to guarantee strong statistical power, 428 individuals were selected as the final sample size after accounting for a 10% non-response rate.

Data collection

Data were collected using a systematic questionnaire and a review of patients' medical records. Obesity prevalence, the primary focus of the study, was defined using the WHO BMI classification (BMI ≥ 30 kg/m²). Additional demographic and clinical data were documented, including age, gender, surgical type (elective or emergency), and comorbidities (e.g., hypertension, diabetes, cardiovascular disease, and chronic respiratory conditions).

To ensure a comprehensive analysis, further variables were recorded. Nutritional status, assessed using parameters like serum albumin levels, smoking history (current or former smoker), and alcohol consumption, was documented. Preoperative functional status, including frailty scores and mobility limitations, was also evaluated. Data on the type and complexity of surgery (minor, major, laparoscopic, or open), anesthetic techniques (regional or general), and intraoperative factors, such as estimated blood loss, were collected.

Postoperative outcomes included complications such as wound infections, respiratory and cardiovascular issues, VTE, renal complications, postoperative bleeding, hernia formation, and gastrointestinal problems. ICU admissions, reoperations, mortality rates, and durations of hospital stay and recovery were also meticulously recorded. These factors were incorporated to provide a holistic view of the impact of obesity on surgical outcomes.

Statistical analysis

Data were analyzed using IBM SPSS Statistics for Windows, Version 25 (Released 2017; IBM Corp., Armonk, NY, USA). Descriptive statistics were used to summarize demographic and clinical characteristics. Obesity prevalence was calculated as a proportion of the study population. Associations between obesity and postoperative complications were examined using t-tests for continuous variables and Chi-square tests for categorical variables, with a p-value < 0.05 considered statistically significant. Subgroup analyses were conducted to evaluate the relationship between obesity and outcomes within surgical type (elective or emergency) and surgical site classification (clean, clean-contaminated, contaminated/septic). Multivariate analyses were performed to adjust for confounders, including age, comorbidities, surgical complexity, and emergency versus elective status, ensuring that the independent effect of obesity on postoperative complications was accurately assessed.

Ethical approval

The Institutional Review Board gave their approval for the research (714/LRH/MTI). Before any data were collected, all patients gave their informed consent, and patient confidentiality was maintained.

## Results

Table 1 summarizes the demographic and clinical characteristics of 428 patients undergoing abdominal surgery. Among them, 245 were male (57.24%) and 183 were female (42.76%), indicating a slight male predominance. The age distribution shows that the largest group consisted of patients aged 31 to 40 years (112 patients, or 26.17%), followed closely by those aged 41 to 50 years (108 patients, or 25.23%), highlighting a prevalence of middle-aged individuals in this surgical population. In terms of BMI, 151 patients were classified as obese (35.28%), 132 as overweight (31.54%), and 142 as having normal weight (33.18%), emphasizing the need for weight management considerations in preoperative assessments. Additionally, 267 patients underwent elective surgery (62.34%), while 161 underwent emergency procedures (37.66%). Common comorbidities included hypertension in 107 patients (25%), diabetes in 89 patients (20.79%), cardiovascular disease in 47 patients (10.98%), and chronic respiratory illness in 39 patients (9.11%). These findings underscore the importance of thorough preoperative evaluations for patients with these health conditions.

**Table 1:** Demographic and Clinical Characteristics of Patients Undergoing Abdominal Surgery (N = 428)

Characteristics	Sub-groups	n (%)
Age (Years)	18-30	85 (19.86)
	31-40	112 (26.17)
	41-50	108 (25.23)
	51-60	80 (18.69)
	>60	43 (10.05)
Gender	Male	245 (57.24)
	Female	183 (42.76)
Body Mass Index (BMI)	Normal Weight (18.5-24.9)	142 (33.18)
	Overweight (25-29.9)	135 (31.54)
	Obese (≥30)	151 (35.28)
Nutritional Status	Albumin Levels < 3.5 g/dL	42 (9.82)
	Preoperative Malnutrition	63 (14.72)
Smoking History	Current Smokers	72 (16.85)
	Former Smokers	89 (20.79)
Alcohol Consumption	No Alcohol Consumption	187 (43.71)
	Moderate Alcohol Consumption (≤3 Drinks/Day)	115 (26.89)
	Heavy Alcohol Consumption (>3 Drinks/Day)	126 (29.40)
Preoperative Functional Status	Independent (No Mobility Limitations)	318 (74.43)
	Mobility Limitations/Frailty Scores Present	110 (25.57)
Anesthetic Techniques	General Anesthesia	378 (88.47)
	Regional Anesthesia	50 (11.53)
Type of Surgery	Elective Surgery	267 (62.38)
	Emergency Surgery	161 (37.62)
Intraoperative Blood Loss	<500 mL	365 (85.34)
	≥500 mL	63 (14.66)
Comorbidities	Diabetes Mellitus	89 (20.79)
	Hypertension	107 (25.00)
	Cardiovascular Disease	47 (10.98)
	Chronic Respiratory Disease	39 (9.11)
	Other Comorbidities (e.g., Chronic Kidney Disease)	55 (12.85)

Table 2 shows the surgical outcomes and postoperative complications experienced by the 428 patients. Among them, 64 patients (14.95%) developed wound infections, while 38 patients (8.88%) required reoperations, and 52 patients (12.15%) were admitted to the ICU. The mortality rate was nine patients (2.10%), indicating some risk associated with the procedures. The majority of patients, 337 (78.74%), had hospital stays of seven days or fewer, reflecting a generally favorable recovery trend, while 91 patients (21.26%) stayed longer. Various postoperative complications were noted, including respiratory problems in 58 patients (13.55%), cardiovascular issues in 44 patients (10.28%), VTE in 27 patients (6.31%), renal problems in 26 patients (6.07%), postoperative bleeding in 33 patients (7.71%), hernia formation in 21 patients (4.91%), and gastrointestinal complications in 40 patients (9.35%). These findings highlight the need for ongoing postoperative monitoring and management due to the range of complications reported.

**Table 2:** Surgical Outcomes and Postoperative Complications (N = 428)

Variable		Number of Patients, n (%)
Surgical Outcomes	Wound Infection	64 (14.95)
	Re-operation	38 (8.88)
	ICU Admission	52 (12.15)
	Mortality	9 (2.10)
Length of Hospital Stay	≤7 Days	337 (78.74)
	>7 Days	91 (21.26)
Postoperative Complications	Venous Thromboembolism (VTE)	27 (6.31)
	Respiratory Complications	58 (13.55)
	Cardiovascular Complications	44 (10.28)
	Renal Complications	26 (6.07)
	Postoperative Bleeding	33 (7.71)
	Hernia Formation	21 (4.91)
	Gastrointestinal Complications	40 (9.35)

Table 3 shows the relationship between obesity and various postoperative complications, categorized into infectious, gastrointestinal, cardiovascular and pulmonary, and renal and other complications. Obese patients had higher rates of infectious complications, with 36 patients (23.84%) affected by wound infections compared to 28 non-obese patients (10.11%). Additionally, gastrointestinal complications were observed in 21 obese patients (13.91%) compared to 19 non-obese patients (6.86%), and cardiovascular and pulmonary complications included VTE in 18 obese patients (11.92%) versus nine non-obese patients (3.25%), and respiratory complications in 27 obese patients (17.88%) compared to 31 non-obese patients (11.19%). Obese patients also experienced more renal and other complications, with renal issues observed in 14 obese patients (9.27%) compared to 12 non-obese patients (4.33%). Postoperative bleeding, hernia formation, and ICU admissions did not show significant differences between the two groups. Furthermore, obese patients had a higher likelihood of prolonged hospital stays (>7 days), with 52 patients (34.44%) experiencing extended stays compared to 39 non-obese patients (14.08%). These findings suggest that obesity significantly impacts surgical outcomes, underscoring the need for targeted interventions for obese patients to improve postoperative recovery and prevent complications.

**Table 3:** Association Between Obesity and Postoperative Complications (N = 428) X² denotes Chi-square. Adjusted Odds Ratio (AOR) was calculated to account for potential confounders such as comorbidities, surgical complexity, and patient demographics. A 95% Confidence Interval (CI) shows the range within which the true odds ratio is likely to fall, reflecting the precision of the estimate. Adjusted p-value provides a refined significance level after accounting for confounding variables.

Category	Complication	Obese (n = 151)	Non-obese (n = 277)	p-value	X²	Adjusted Odds Ratio (AOR)	95% Confidence Interval (CI)	Adjusted p-value
Infectious Complications	Wound Infection	36 (23.84%)	28 (10.11%)	0.001	10.16	2.89	1.61-5.19	0.001
	Gastrointestinal Complications	21 (13.91%)	19 (6.86%)	0.015	5.73	2.16	1.12-4.17	0.021
Cardiovascular and Pulmonary	Venous Thromboembolism (VTE)	18 (11.92%)	9 (3.25%)	0.002	9.04	3.24	1.44-7.28	0.005
	Respiratory Complications	27 (17.88%)	31 (11.19%)	0.045	4.06	1.71	1.01-2.89	0.048
	Cardiovascular Complications	20 (13.25%)	24 (8.66%)	0.106	2.46	1.56	0.86-2.85	0.137
Renal and Other Complications	Renal Complications	14 (9.27%)	12 (4.33%)	0.037	4.31	2.23	1.03-4.85	0.043
	Postoperative Bleeding	12 (7.95%)	21 (7.58%)	0.88	0.03	1.04	0.49-2.21	0.902
	Hernia Formation	11 (7.28%)	10 (3.61%)	0.085	3.07	2.08	0.91-4.74	0.085
	Prolonged Hospital Stay (>7 Days)	52 (34.44%)	39 (14.08%)	<0.001	22.05	3.22	1.99-5.21	<0.001
	ICU Admission	23 (15.23%)	29 (10.47%)	0.162	1.98	1.54	0.85-2.80	0.168

The majority of participants were older, with an average age of 47.32 ± 11.54 years versus 43.10 ± 12.68 years. The obese patients experienced longer recovery periods of 11.58 ± 5.67 days, extended hospital stays averaging 9.62 ± 4.75 days, and increased surgical durations of 124.35 ± 35.82 minutes, compared to 108.65 ± 29.44 minutes for non-obese patients. The relationship between obesity and various postoperative complications shows that obese patients face higher rates of issues such as wound infections (23.84% in obese versus 10.11% in non-obese) and VTE (11.92% in obese versus 3.25% in non-obese), suggesting that obesity significantly impacts surgical outcomes. The association between obesity and prolonged hospital stays indicates that this group may require additional resources and support during their recovery. Interestingly, not all complications were influenced by obesity, implying that other factors may also contribute to postoperative outcomes. These findings highlight the complexities of managing obesity in a surgical context and underscore the need for comprehensive postoperative care strategies to ensure optimal recovery (Table 4).

**Table 4:** t-tests for Continuous Variables (N = 428) * indicates independent t-tests

Variable	Obese (Mean ± SD)	Non-obese (Mean ± SD)	p-value*	t-value
Age (Years)	47.32 ± 11.54	43.10 ± 12.68	0.001	3.35
Hospital Stay (Days)	9.62 ± 4.75	6.12 ± 3.81	<0.001	5.65
Surgery Duration (Minutes)	124.35 ± 35.82	108.65 ± 29.44	0.003	3
Postoperative Recovery (Days)	11.58 ± 5.67	8.73 ± 4.25	0.002	3.13

## Discussion

The primary aim of this study was to investigate the relationship between obesity and various postoperative complications among patients undergoing abdominal surgery. Our findings underscore the significant role that obesity plays in increasing the risk of several postoperative complications, emphasizing the need for tailored preoperative and postoperative care strategies for obese patients to optimize outcomes and recovery.

In our study, 35.28% of patients were classified as obese, with a mean BMI of 34.8 ± 3.2 kg/m², reflecting the distinct characteristics of this subgroup. This percentage aligns with the growing global prevalence of obesity and its recognized impact on surgical outcomes. Notably, the proportion of obese patients in our cohort is consistent with the 33% reported by Wallace et al. in a previous study among U.S. surgical patients [16], further highlighting that obesity is a widespread concern in surgical populations. These findings emphasize the critical need for targeted interventions to mitigate the adverse effects of obesity on surgical outcomes and recovery.

Our analysis demonstrated that obese patients had significantly higher rates of wound infections, VTE, respiratory complications, and renal complications compared to non-obese patients. Specifically, obese patients experienced wound infections at a rate of 23.84%, compared to 10.11% in non-obese patients, which is consistent with other research showing that surgical site infections are almost twice as common in obese individuals [17]. This increased risk is likely due to factors such as impaired wound healing, increased tissue tension, and the presence of comorbidities like diabetes, which was present in 20.79% of our patients.

Similarly, our study found that the incidence of VTE was significantly higher in obese patients (11.92%) than in non-obese patients (3.25%). This finding mirrors previous studies that have shown that obesity leads to increased intra-abdominal pressure and reduced mobility, both of which elevate the risk of VTE, particularly after major abdominal surgeries [18]. These results underscore the importance of proactive measures to prevent thromboembolic events in obese surgical patients.

Respiratory complications were also more prevalent in obese patients, with 17.88% of obese patients experiencing postoperative respiratory issues, compared to 11.19% of non-obese patients. Obesity adversely affects pulmonary function, leading to decreased lung volumes and an increased risk of atelectasis and hypoxemia, which are particularly problematic in the postoperative period. This finding is in agreement with research by Doyle et al. [19], which demonstrated an increased risk of respiratory complications in obese patients following abdominal surgery.

Our study further found that obese patients required longer recovery periods, with an average of 11.58 ± 5.67 days, compared to 8.73 ± 4.25 days for non-obese patients. In addition, obese patients had longer hospital stays (9.62 ± 4.75 days versus 6.12 ± 3.81 days), both of which are consistent with findings from other studies that have shown that obesity delays recovery and extends hospital stays due to increased complication rates and slower healing processes [20]. The longer surgical duration observed in obese patients (124.35 ± 35.82 minutes versus 108.65 ± 29.44 minutes) is consistent with the technical challenges involved in performing abdominal surgeries on obese patients, including larger tissue mass and difficulty in surgical access [21].

Study limitations and future research

Our study provides valuable insights into the relationship between obesity and postoperative complications; however, it has certain limitations. The single-center design may limit the generalizability of our findings to other populations or diverse healthcare settings. Additionally, the cross-sectional nature of the study restricts our ability to assess long-term postoperative outcomes and complications. While we analyzed a comprehensive range of variables, factors such as the severity of comorbid conditions and surgical complexity were not fully adjusted for, which could introduce potential biases.

To minimize bias and improve the robustness of our findings, we implemented several strategies. First, we used a standardized data collection process, including a systematic questionnaire and a review of medical records, to ensure consistency and accuracy. Second, we employed subgroup and multivariate analyses to account for confounding variables, such as patient age, comorbidities, and type of surgery. These efforts helped isolate the independent effect of obesity on postoperative outcomes. Third, we included both emergency and elective surgeries across a wide range of BMI categories, providing a more diverse representation of surgical cases. Finally, to reduce sampling bias, we calculated a statistically powered sample size and recruited participants systematically throughout the study period.

Future research should build on these steps by conducting prospective, multicenter studies to enhance generalizability. Incorporating advanced multivariable analyses and adjusting for additional confounders, such as the severity of comorbid conditions and the complexity of surgical procedures, can provide a deeper understanding of obesity’s impact on both immediate and long-term surgical outcomes. Additionally, while our study defined obesity based on BMI, we acknowledge that BMI does not capture differences in fat distribution, muscle mass, or body composition. Future studies should consider incorporating alternative measures of body fat, such as waist-to-hip ratio or body composition analysis, to offer a more comprehensive understanding of how obesity influences surgical outcomes. Furthermore, although we focused primarily on obesity, it is crucial to recognize that other factors, such as the type of surgery and the presence of additional comorbidities, may also contribute to postoperative complications. Our findings suggest that, while obesity is a significant risk factor, a holistic approach that considers all aspects of patient health, including comorbidities and surgical complexity, is essential for optimizing surgical outcomes.

## Conclusions

This study highlights the significant prevalence of obesity among patients undergoing abdominal surgery, with over one-third classified as obese and presenting with notable comorbidities, such as hypertension, diabetes, and cardiovascular disease. Obesity was strongly associated with increased postoperative risks, including wound infections, VTE, respiratory complications, longer surgical durations, extended hospital stays, and prolonged recovery times. These findings underscore the importance of comprehensive preoperative assessments and targeted interventions to mitigate the compounded risks of obesity and its associated health conditions in surgical patients.

While the study primarily aimed to document obesity prevalence, it also revealed its substantial impact on surgical outcomes. However, the multifactorial nature of postoperative complications warrants cautious interpretation, as not all adverse outcomes were directly attributable to obesity. Future studies should validate these findings across diverse populations, investigate long-term implications, and explore comprehensive care protocols tailored to optimize surgical outcomes for obese patients. Such efforts are critical to enhancing patient safety and recovery in surgical settings.
